# Functional Exercise

**Published:** 1892-04

**Authors:** 


### FUNCTIONAL EXERCISE.

Extract from an address delivered by Sir James Crichton Browne:

It seems a physiological law that the functions of the body must be kept in exercise in order to maintain its efficacy, and it is as true of the body as of the mill, or of any other machine, that it will rust out from disuse sooner than wear out by employment. The fact is constantly observed in persons engaged in commercial pursuits, who retire at the age of sixty, and then fall into rapid decay, while professional men remaining at work preserve their vigor often for another twenty years.

There is no reason why old age should not be as happy and enjoyable as any other period of life. If old persons be asked as to their consciousness of age, they will all, with one consent, declare that there exists nothing of the kind. An old person has a knowledge of his age in the same way as his friends; he sees it by looking in the mirror, by remembrance of past events, or the loss of contemporaries ; but he is not constantly carrying about with him the conviction or feeling that he is old. He is thus still able to occupy himself in the business and pleasures of life.

Buffon spoke of his green old age as one of the happiest periods of his life, although the kind of pleasures then experienced are, of course, different from those of youth. And even when decay comes, and a man is becoming free from the remembrance of all earthly things, then, as Sir James Paget says (and no better example could be found of full mental activity by continued work), it may be so ordered on purpose that the spirit may be invigorated and undisturbed in the contemplation of the brightening future.

It is a sad thing to see the nerve centres decay with a corresponding weakness of body and mind ; but it is still sadder to witness with a wrinkling of the skin a corresponding shrinking of the brain, allowing vanity, and some of the weakly passions which had been kept in suppression, to come again to the fore. How different is the spectacle when the organ is kept in its integrity by constant use, and the mental faculties preserved in all their pristine force. We have only to look around and to see our poets, bishops, judges, ministers of state and medical men, long-lived and still in mental vigor while working at their respective avocations.

It is clear that hard work does not kill. The toil, however, must be genial and diversified. The man of business often has no occupation besides his bread-winning, whereas, a medical man has a variety of subjects to interest him.

A speaker at a recent International Congress showed by experiments upon school children, when three or four examples in arithmetic were given in succession, that each showed an inferiority to the previous one, both in correctness and as regards the time in which it was completed. The one faculty employed was gradually exhausted—a fresh piece of evidence showing the necessity of diversity of work. In the treatment of persons with mental trouble or worry, the very worst method is to rely too much on what is called rest, meaning thereby leaving the patient without other employment than to brood over his sorrows. True rest to the mind is only to be obtained by the occupation of other faculties roused into action by new surroundings.

A writer, speaking of old age, in reference to the decease of an eminent barrister, also maintained that the highest faculties are kept keen by constant exercise, and the brain vigorous by constant action and renewal. The understanding has often been in the highest perfection in quite advanced old age, and that has been the best period of human life. It is the time when the rage and storm of passion have died away, when the jealousies and cares of a career have ceased and been forgotten, when memory lingers upon all that is bright and charming in the past, and when hope scatters her most glowing tints over a fast approaching future, or we are able to see in old age glimpses of the truth that its chief glory consists not in the remembrance of feats of prowess, nor in the egotistic exercise of power, but in the conquest of peevish weakness, in the brightness of hope, and in the distribution of happiness around. Depend upon it, the best antiseptic against senile decay is an active interest in human affairs, and those keep young longest who love most and study most. Cato learned Greek at eighty, and Sophocles wrote his " Lives of Men " after reaching three score years and ten.

